# Posterior cerebral artery dissection after excessive caffeine consumption in a teenager

**DOI:** 10.1016/j.radcr.2022.02.035

**Published:** 2022-04-12

**Authors:** Nikolaos Staikoglou, Aspasia Polanagnostaki, Viktoria Lamprou, Evangelos Chartampilas, Evangelos Pavlou, Thomas Tegos, Stephanos Finitsis

**Affiliations:** a Radiology Department, University General Hospital of Thessaloniki AHEPA, Thessaloniki, Kentriki Makedonia 546 21, Greece; b Second Department of Pediatrics, University General Hospital of Thessaloniki AHEPA, Thessaloniki 546 21, Greece; c First Department of Neurology, University General Hospital of Thessaloniki AHEPA, Thessaloniki 546 21, Greece

**Keywords:** Dissection, Posterior cerebral artery, Pediatric stroke, Caffeine

## Abstract

Arterial ischemic stroke is a rare but significant cause of neurological deficits in childhood. Even though there is a variety of risk factors, identifying the etiology can sometimes be a hard diagnostic challenge. Arteriopathies in general, and more specifically, arterial dissection is one of the uncommon pathologies that can cause incidents of pediatric stroke. We report a rare case of a young adolescent with posterior cerebral artery dissection after excessive consumption of caffeine, contained in energy drinks, only hours before the onset of neurological symptoms. A complete neuroimaging evaluation (MRI, intracranial US and digital subtraction angiography) at the admission and during the follow-ups supported the diagnosis of arterial dissection possibly caused by caffeine overconsumption.

## Introduction

Arterial ischemic stroke (AIS) is an uncommon but important cause of acquired neurological deficits in childhood. Risk factors in children are different from those in adults. Multiple risk factors may coexist, with arteriopathy being the most important [1]. Among non – inflammatory causes of arteriopathy, dissection of cerebral vessels is responsible for 7,5%-20% of pediatric AIS. Vertebral and basilar arteries are most often involved and, less commonly, the carotid and middle cerebral arteries. Dissection of the posterior cerebral artery (PCA) is uncommon across the entire age spectrum, especially among children. The primary etiology of PCA dissection is trauma and, less often, other vasogenic factors [2]. We report a case of posterior cerebral artery (PCA) dissection in a child after excessive energy drink consumption. To the best of our knowledge, this is the first case documented.

## Case description

**Fig. 1:**
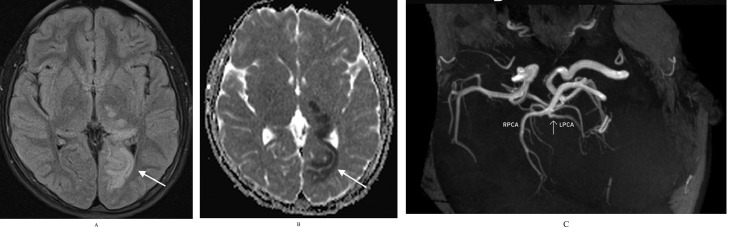
MRI on Day 1. (A) There exists a high signal in the cortex of the inner surface of the left occipital lobe and in the left thalamus (arrow). (B) The lesions have a low signal on the ADC map characteristic for ischemia. C) On a 3D-TOF oblique projection, there is a narrowing at the P1-P2 junction while the P4 segment is not apparent.

**Fig. 2:**
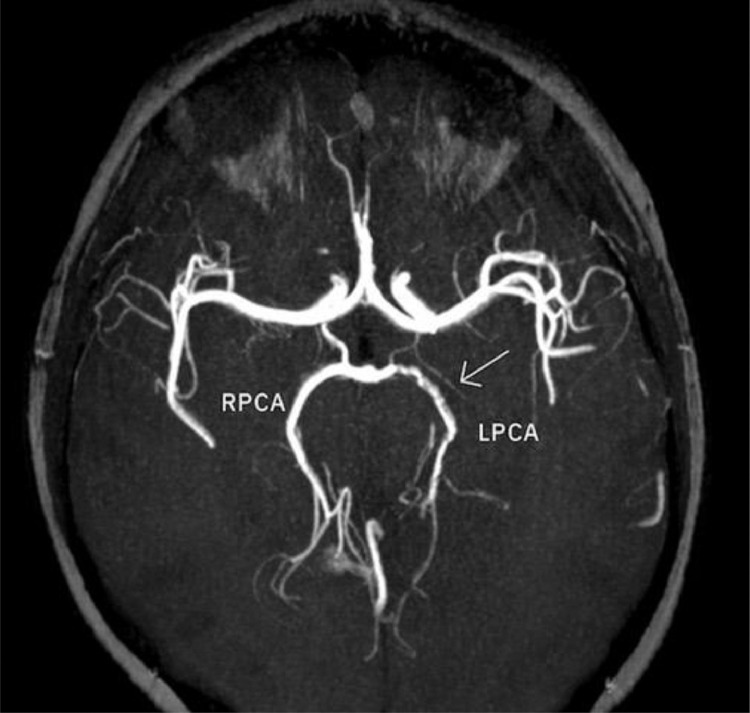
Imaging on Day 4 shows the typical “string and pearl” sign of the left P2 and P3 PCA segments on 3D-TOF sequences.

**Fig. 3:**
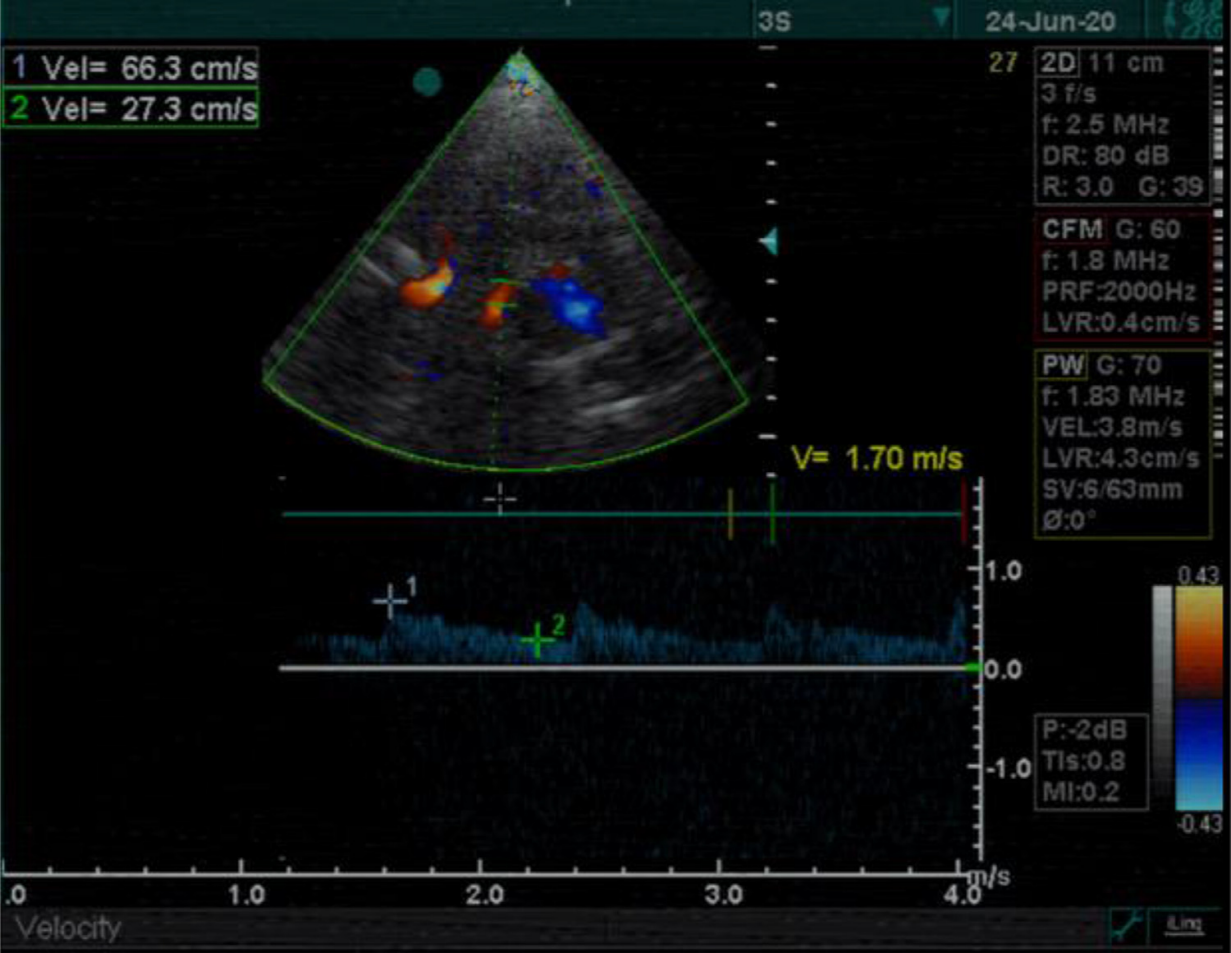
Intracranial Doppler ultrasonographic examination at the level of the left P1-P2 segment shows an attenuated flow pattern compared to the contralateral side (not shown).

**Fig. 4:**
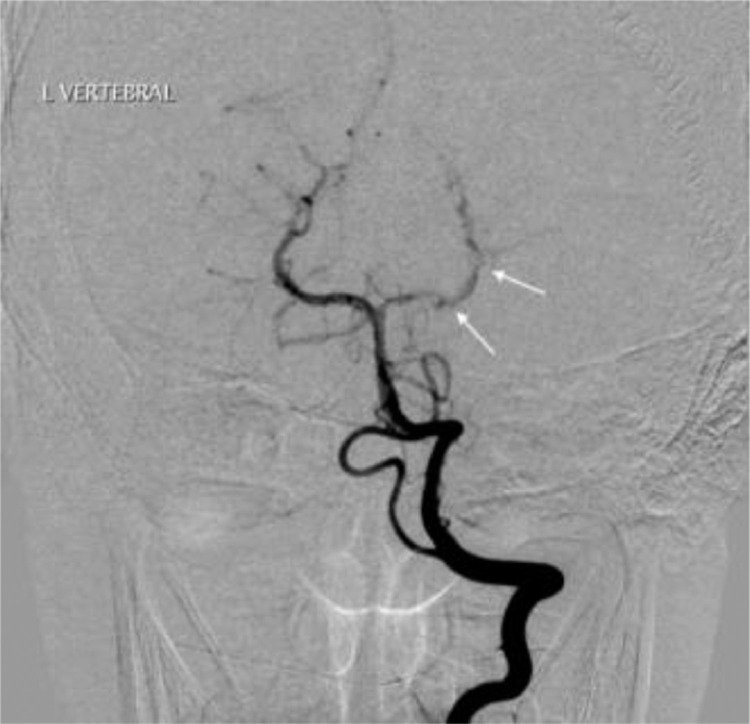
Digital subtraction angiography on Day 11 demonstrates the typical “string and pearl” sign at the level of the left P1-P2 junction and distally.

**Fig. 5:**
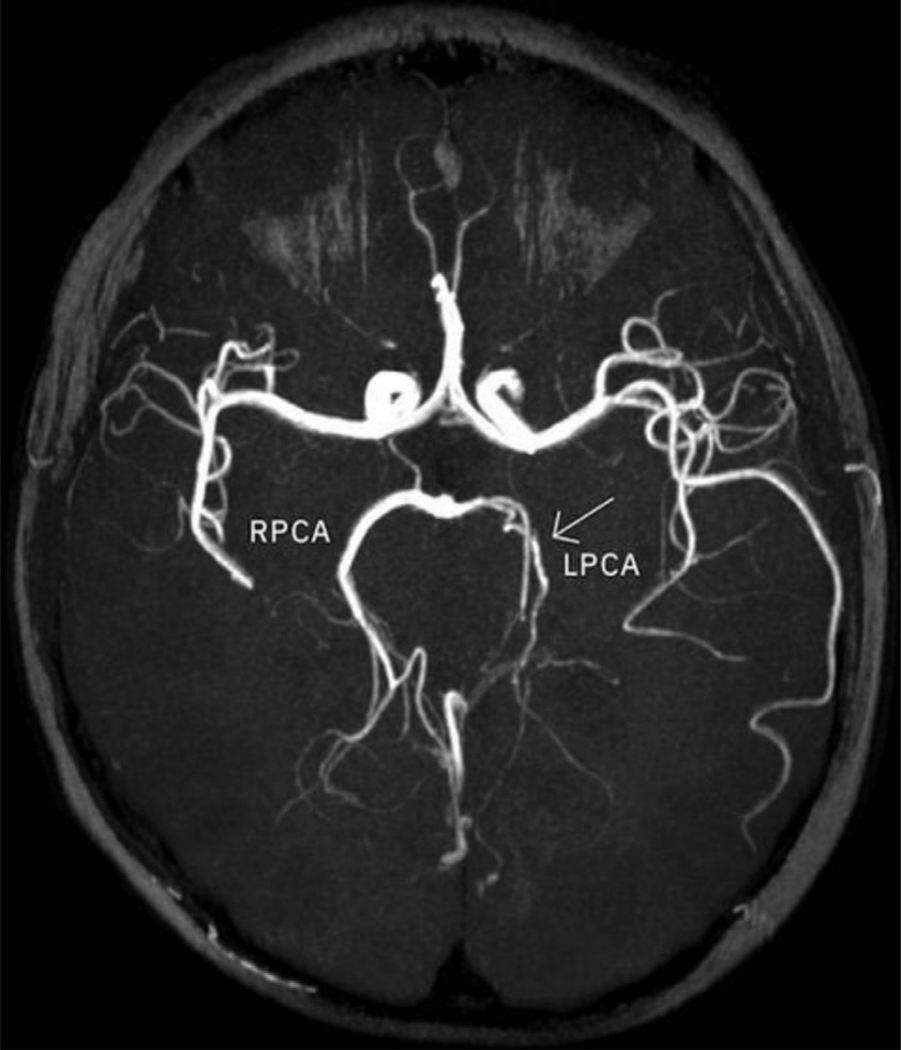
Imaging on day 30 shows residual narrowing of the left PCA.

A 14-year-old boy with no past medical history was admitted to the emergency department with dysarthria, headache, mild right-hand weakness, hypesthesia, and right optic field deficits. The patient reported consuming 2 liters of energy drink (high caffeine intake) during the previous 10 hours. Blood pressure was 190/120 mmHg and the heart rate 116/min. Qualitative toxicology tests were positive for caffeine. A brain CT scan was performed right after the admission, with unremarkable findings. 3 days later, a brain MRI/MRA scan revealed an area of high T2 signal and restricted diffusion (high signal in DWI and low ADC) in parts of the left thalamus, splenium, and inner occipital lobe (Fig. 1). On the MRA-TOF sequences, the P2 segment of the left PCA appeared narrowed, while the P4 segment had no flow signal. Anticoagulation therapy was immediately initiated. 3 days later, a second MRI scan showed a typical “string and pearls” sign of the left P2 and P3 segments (Fig. 2). The cardiac and carotid ultrasonographic exams, the EEG, and the rest of the lab tests were unremarkable. An intracranial doppler was also performed but did not indicate any thrombus or vasospasm (Fig. 3). Based on the patient history of excessive caffeine consumption, elevated blood pressure at admission, and the “string and pearl” sign on MR angiography, the diagnosis was brain ischemia secondary to dissection of the PCA. Digital subtraction angiography performed 11 days after symptom onset confirmed the diagnosis (Fig. 4). The patient was discharged 2 weeks later with no neurological deficit, and a new MR Angiography showed improvement of PCA vessel wall lesions (Fig. 5).

## Discussion

Childhood arterial ischemic stroke (AIS) is an important cause of acquired brain injury in young ages with severe social and economic impact [3,4]. Pediatric stroke etiologies differ from adult stroke, and their understanding is limited. 50% of cases present multiple risk factors, while another 17% remain diagnosed as idiopathic [5]. The contribution of congenital and inherited abnormalities to intracranial arterial dissections is unknown [7]. Almost one-third of children are finally diagnosed with focal or systematic arteriopathy. Therefore, vascular imaging has a central role in the correct etiological diagnosis [8,9].

Extracranial dissections are a common etiology of pediatric stroke. Intracranial dissections are rare and probably underdiagnosed as they may appear as simple vessel occlusions [10]. The mechanism of brain ischemia is more likely to be hypoperfusion for intracranial dissections and thromboembolism for extracranial carotid and vertebral artery dissections. Although arterial hypertension is commonly associated with extracranial carotid dissection, few patients with posterior circulation dissection have hypertension [6].

PCA dissections most commonly occur near the P1-P2 junction (close to the free border of the tentorium cerebelli). The most common symptom is headache (mainly in the occipital and posterior cervical regions), and imaging usually demonstrates cerebral ischemia or concomitant subarachnoid hemorrhage in 25% of cases [7]. Imaging with computed tomography or MR angiography may establish the diagnosis but digital subtraction angiography remains the gold standard. Imaging demonstrates narrowing of the vascular lumen secondary to the subintimal hematoma (string sign), with or without segmental dilation proximal or distal to the stenosis (string and pearl sign). PCA dissection has a more benign clinical course and prognosis than other intracranial dissections [8].

The present case is unique because the etiology of intracranial dissection was consuming excessive caffeine (included in energy drinks) by a young patient in a short timeframe before the onset of symptoms. The patient had no other risk factors or any other comorbidities. Zacher et al. reported a case of coronary artery dissection of similar etiology in a young adult [9]. Caffeine consumption usually aims at central nervous system arousal, but its effects on the cardiovascular system are manifold; physiologically, caffeine causes coronary and cerebral vasoconstriction, smooth muscle relaxation, skeletal muscle stimulation, cardiac chronotropic and inotropic effects, and reduction of insulin sensitivity [10]. Moreover, “energy drinks” may contain other ingredients, such as guarana, one of the highest caffeine-containing plants known.

As the consumption of “energy drinks” appears to expand to younger age groups, cerebral ischemia and dissection secondary to overconsumption should be included in the differential diagnosis of pediatric ischemic stroke.

## References

[1] deVeber G.A., Kirton A., Booth F., Yager J., Wirrell E., Wood E. (2017). Epidemiology and outcomes of arterial ischemic stroke in children: the canadian pediatric ischemic stroke registry. Pediatr. Neurol..

[2] Bernard T., Manco-Johnson M., Warren L., MacKay M., Ganesan V., DeVeber G. (2012). Towards a consensus-based classification of childhood arterial ischemic stroke. Stroke.

[3] Lo W., Khaled Z., Ponnappa K., Allen A., Chisolm D., Tang M. (2008). The Cost of Pediatric Stroke Care and Rehabilitation. Stroke.

[4] Mardee G., Anne G., Vicki A., T M.M. (2016). Outcome in Childhood Stroke. Stroke.

[5] Mallick A., Ganesan V., Kirkham F., Fallon P., Hedderly T., McShane T. (2014). Childhood arterial ischemic stroke incidence, presenting features, and risk factors: a prospective population-based study. Lancet Neurol.

[6] M. S. Berger, C. B. Wilson, Intracranial dissecting aneurysms of the posterior circulation, *J. Neurosurg.*, 61, 5, 882–894, doi:10.3171/jns.1984.61.5.0882. 6491734

[7] Lazinski D., Willinsky R.A., TerBrugge K., Montanera W. (2000). Dissecting aneurysms of the posterior cerebral artery: angioarchitecture and a review of the literature. Neuroradiology.

[8] Taqi M., Lazzaro M., Pandya D., Badruddin A., Zaidat O. (2011). Dissecting aneurysms of posterior cerebral artery: clinical presentation, angiographic findings, treatment, and outcome. Front Neurol.

[9] Zacher J., May E., Horlitz M., Pingel S. (2018). Binge drinking alcohol with caffeinated ‘energy drinks’, prolonged emesis and spontaneous coronary artery dissection: a case report, review of the literature and postulation of a pathomechanism. Clin. Res. Cardiol..

[10] Seifert S.M., Schaechter J.L., Hershorin E.R., Lipshultz S.E. (2011). Health effects of energy drinks on children, adolescents, and young adults. Pediatrics.

